# A Deep Learning Model for Detecting Cage-Free Hens on the Litter Floor

**DOI:** 10.3390/ani12151983

**Published:** 2022-08-05

**Authors:** Xiao Yang, Lilong Chai, Ramesh Bahadur Bist, Sachin Subedi, Zihao Wu

**Affiliations:** 1 Department of Poultry Science, College of Agricultural & Environmental Sciences, University of Georgia, Athens, GA 30602, USA; 2 Department of Computer Science, Franklin College of Arts and Sciences, University of Georgia, Athens, GA 30602, USA

**Keywords:** egg production, cage-free system, precision farming, deep learning algorithms

## Abstract

**Simple Summary:**

Real-time and automatic detection of chickens such as laying hens and broilers is the cornerstone of precision poultry farming. For laying hens, it is more challenging under cage-free conditions comparing to caged systems. In this study, we developed a deep learning model (*YOLOv5x-hens*) to monitor hens’ behaviors in cage-free facilities. More than 1000 images were used to train the model and an additional 200 images were adopted to test it. The newly developed *YOLOv5x-hens* was tested with stable performances in detecting birds under different lighting intensities, angles, and ages over 8 weeks. According to further data analysis, the model performed efficiently in real-time detection with an overall accuracy more than 95%, which is the key step for the tracking of individual birds for evaluation of production and welfare. However, there are still some limitations of the current version of the model. Error detections were caused by highly overlapped stock, uneven light intensity, and images occluded by equipment (i.e., drinking lines and feeders). Future research is needed to address those issues for a higher detection rate. The current study provides technical basis for developing a machine vision system for tracking individual birds for evaluation of animals’ behaviors and welfare status in commercial cage-free houses.

**Abstract:**

Real-time and automatic detection of chickens (e.g., laying hens and broilers) is the cornerstone of precision poultry farming based on image recognition. However, such identification becomes more challenging under cage-free conditions comparing to caged hens. In this study, we developed a deep learning model (*YOLOv5x-hens*) based on *YOLOv5*, an advanced convolutional neural network (CNN), to monitor hens’ behaviors in cage-free facilities. More than 1000 images were used to train the model and an additional 200 images were adopted to test it. One-way ANOVA and Tukey HSD analyses were conducted using JMP software (JMP Pro 16 for Mac, SAS Institute, Cary, North Caronia) to determine whether there are significant differences between the predicted number of hens and the actual number of hens under various situations (i.e., age, light intensity, and observational angles). The difference was considered significant at *p* < 0.05. Our results show that the evaluation metrics (Precision, Recall, F1 and mAP@0.5) of the *YOLOv5x-hens* model were 0.96, 0.96, 0.96 and 0.95, respectively, in detecting hens on the litter floor. The newly developed *YOLOv5x-hens* was tested with stable performances in detecting birds under different lighting intensities, angles, and ages over 8 weeks (i.e., birds were 8–16 weeks old). For instance, the model was tested with 95% accuracy after the birds were 8 weeks old. However, younger chicks such as one-week old birds were harder to be tracked (e.g., only 25% accuracy) due to interferences of equipment such as feeders, drink lines, and perches. According to further data analysis, the model performed efficiently in real-time detection with an overall accuracy more than 95%, which is the key step for the tracking of individual birds for evaluation of production and welfare. However, there are some limitations of the current version of the model. Error detections came from highly overlapped stock, uneven light intensity, and images occluded by equipment (i.e., drinking line and feeder). Future research is needed to address those issues for a higher detection. The current study established a novel CNN deep learning model in research cage-free facilities for the detection of hens, which provides a technical basis for developing a machine vision system for tracking individual birds for evaluation of the animals’ behaviors and welfare status in commercial cage-free houses.

## 1. Introduction

Daily routine evaluation of chickens (e.g., broilers and layers) is critical for maintaining animals’ health and welfare in commercial poultry houses [1,2,3]. For laying hen production, this is becoming more challenging under cage-free production systems as compared to conventional caged systems because hens can move freely in cage-free houses, where animals have opportunities to perform common natural behaviors [4,5]. In recent years, computer vision has been used to monitor farm animals due to the non-invasive nature of this method. A completed object detection cycle in the computer vision includes observation, diagnostics, and prediction. In using computer vision, visual sensors such as cameras are installed at fixed locations to collect images or videos of animals (i.e., cattle, pigs, and poultry) [6]. Collected data (i.e., images and videos) are fed to diagnostic components (i.e., cloud storage or digital video recorder) for further analysis with machine learning or deep learning models, which need to be specifically programmed to extract object features (e.g., chickens’ profile and body features) and predict the target class, and thus determine the accuracy of the classification.

Deep learning models process images with the self-learning capability, which enables models to perform well in various animal farming environments [6]. Earlier studies have investigated pigs by using deep learning techniques to locate their positions and track their movements. These image processing algorithms showed acceptable accuracy when cameras were installed to collect images of top view animals because the difference in colors between background and animals are clear [7,8]. Similar methods have been used to detect broilers’ behaviors and changes over time in different areas [9,10]. Convolutional neural network (CNN) is one of the most stable and effective techniques in deep learning for animal detection [11,12,13]. The combination of CNN and image processing has been developed to detect chickens. In the previous research, detection of chicken behaviors (i.e., drinking and feeding) has been conducted accurately by two-stage CNN [14,15]. The two-stage CNN method generates whole bounding boxes first and then the detection network determines target objects. Although it functions well in terms of accuracy of classification, each individual component of the two-stage CNN model must be trained separately, and it requires higher computation, and thus slows analysis speed. To enhance real-time detection accuracy, the YOLO (you only look once) model has been developed as a one-stage CNN for object detection. With its end to end training and entire feature maps to predict each bounding box, it performed well on real-time behavior detection of broilers and breeders [16,17]. Ye et al. (2020) used the CNN algorithm (YOLO + multilayer residual module (MRM)) to detect 180,000 white feather broilers per hour [18]. Anlan et al. (2019) developed a YOLOv3 model to detect and locate yellow feather broilers to investigate their heat stress conditions [19]. Zhang et al. (2019) proposed a deep learning model to detect sick broilers simultaneously [20]. However, most of these deep learning methods for poultry detection are focused on broilers. Few studies investigated cage-free layers because it is hard to collect clear images in cage-free houses due to the dusty environment. Deep learning has been tested on target detection for dusty images [21,22]. In fact, that is why the deep learning model is important, because it can detect birds more accurately than our human eyes in chicken houses. The egg industry is shifting to cage-free houses to improve bird welfare, providing enough space for birds to perform their natural behaviors [23], now that all eggs sold in California must come from hens living in cage-free houses [24]. With the increase in cage-free systems in the USA and EU countries, it is critical to develop an automatic method for detecting laying hens on the litter floor of cage-free houses.

The objectives of this study were to: (1) develop a detector for monitoring the real-time number of laying hens in different fixed zones on the floor of cage-free facilities; (2) train the *YOLOv5* model (a newer version of YOLO object detection model) with hens’ images/videos collected at different ages, angles, light intensities, and stock densities; and (3) test the performance of newly developed models (*YOLOv5x-hens*) under various production situations.

## 2. Materials and Methods

### 2.1. Experimental Setup

**Figure 1:**
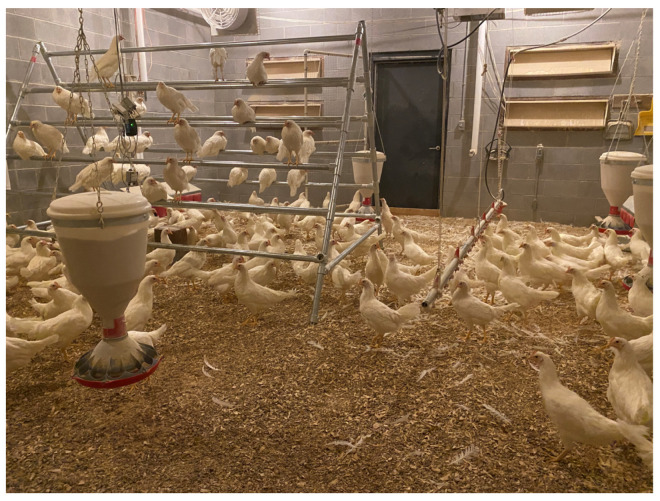
Experiment setup of cage-free research houses.

About 800 one-day-old Hy-Line W-36 chicks are reared evenly in the four rooms, each was measured as 24 ft long, 20 ft wide and 10 ft high (7.3 mL × 6.1 mW × 3 mH), in the University of Georgia (UGA) Poultry Research Center (Athens, GA, USA) (Figure 1). Each room contains six hanging feeders, two drinker kits, and a portable A-frame hen perch to encourage birds’ natural perching behaviors.

The raising environmental conditions were controlled by the automatic system (CHORE-Time Controller, Milford, IN, USA) and the set points were following Hy-Line W-36 commercial layer management guides. The relative humidity (RH) was controlled between 40–60%, air temperature was set at 21–23 °C, light intensity was controlled at 20 lux and lighting period was 19L:5D during the egg laying. The feed was soy-corn manufactured in UGA feed mill every two months to maintain fresh quality and inhibit mildewing. Team members checked the growth and environmental conditions of hens every day as suggested by the UGA Poultry Research Center Standard Operating Procedure Form. The animal use and management were approved by the Institutional Animal Care and Use Committee (IACUC) of the UGA.

### 2.2. Date Acquisition

**Figure 2:**
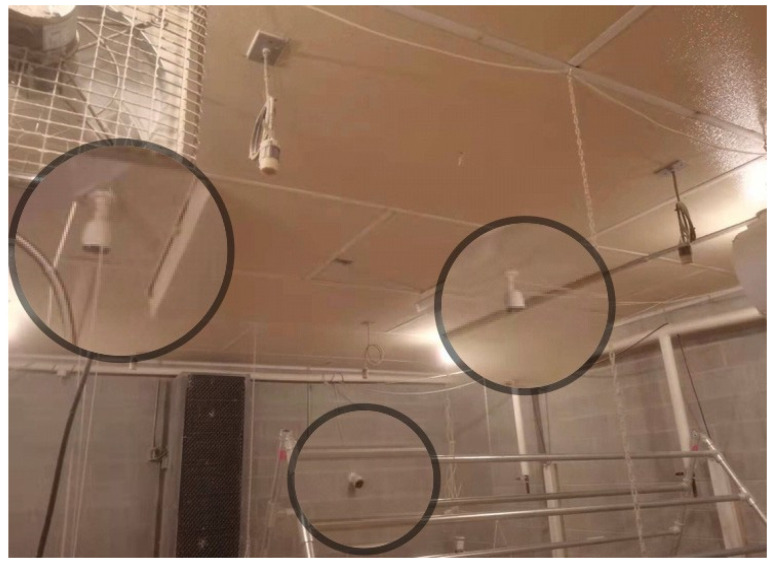
Positions of installed cameras.

Waterproof HD cameras (PRO-1080MSFB, Swann Communications, Santa Fe Springs, CA, USA) were installed on the ceiling and the side wall in each room to collect chickens’ video data (18 frames per second (FPS), 1440 pixels high and 1080 pixels wide) and the installation height of cameras was 3 m (Figure 2). To protect the lens and collect clear video, lens cleaning cloth was used to wipe off dust weekly. Footage data were saved on video recorders (Swann Company, Santa Fe Springs, CA, USA) temporarily on the farm and then transferred to massive hard drives (HDD) (Western Digital Corporation, CA, USA) for safe storage in the data hub in the Department of Poultry Science at UGA.

### 2.3. Data Labeling

**Figure 3:**
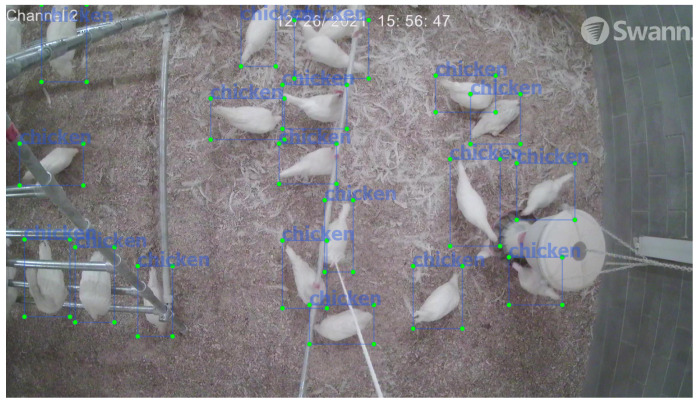
Examples of chicken image labeling.

Videos recorded at birds’ age of 8–16 weeks (as this is a transition period from pullet to layers) were used for data analysis to make sure the method would be applicable for both hens and pullets. Birds’ images were randomly extracted from HDD and converted to JPG format by Free Video to JPG Converter. Total function was selected at the converting process to obtain random pictures. After removing blurred images, 1200 photos were labeled through Labeling Windows_v1.6.1. During labeling operation, chickens with 1/3 or more body contained in the image were labeled by bounding box (Figure 3).

### 2.4. Model Innovation for Detecting Chickens

The *YOLOv5x* model was adapted and innovated by integrating hens’ image information as a new model “*YOLOv5x-hens*” for detecting birds on the litter floor. The *YOLOv5x* model is one of the four most commonly used models for object detection in *YOLOv5* (i.e., *YOLOv5x*, *YOLOv5s*, *YOLOv5m* and *YOLOv5l*) [25]. Compared to the other three models, the *YOLOv5x* model is more powerful and flexible in detecting small-size objects such as chickens due to its enhanced characteristics of depth_multiple, width_multiple, the number of residual network (ResNet) in cross stage partial network (CSPNet), and the amount of convolution kernel (CK). Table 1 shows the differences between the aforementioned models. The *YOLOv5x* model has the best performance among the whole elements, which optimize resolution and capacity of *YOLOv5x* in network. Therefore, the *YOLOv5x* model was adopted and innovated for detecting chickens under sheltered and overlapped situations.

**Figure 4:**
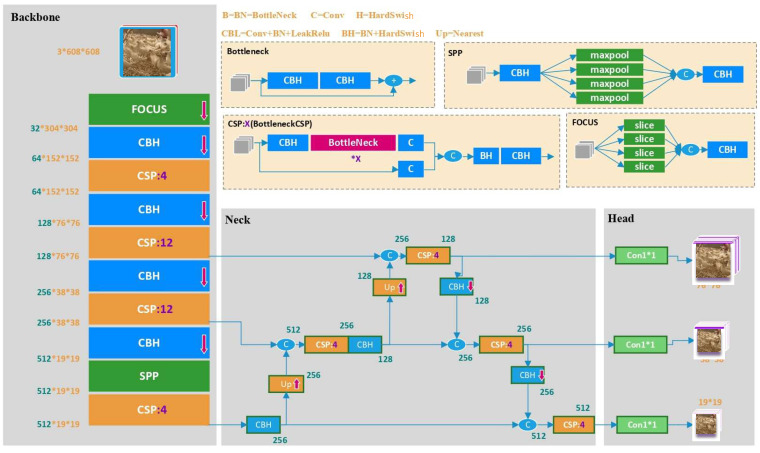
The network structure of *YOLOv5x*.

The network structure is shown in Figure 4. FOCUS means focusing width and height information into channel space. “Conv + Bottle Neck + Hard Swish (CBH)” aimed to extract short-term time features. Cross stage partial (CSP) partitions and merges the feature maps for object detection. Spatial pyramid pooling (SPP) results in fixed-length representations by resampling the feature maps [29].

*Model Backbone*: Four different sub models were developed in the backbone of *YOLOv5x* to extract the basic features of hens. Compared to *YOLOv4*, *YOLOv5x* improved mosaic data enhancement method by adding FOCUS structure, and thus our newly developed model *YOLOv5x-hens* is expected to be more accurate in small object detection [30]. Alphanumeric characters refer to the number of ResNet in distant CSPNet (i.e., CSP:4 indicates that there are four ResNet in this CSPNet).

*Model Neck and Head*: The model of *YOLOv5x-hens* added bottom-up path augmentation by using PAFPN [31], which is a feature pyramid module. The neck utilizes different feature pyramids to recognize the same chicken under diverse sizes and scales. There are three different levels of feature maps at the head phase by a 1 × 1 convolutional layer [32]. This module can maintain chicken’s salient features as well as control the increase in the number of feature maps, so as to decrease the amount of computation required. Finally, three decreased feature maps of same target were used during detection tests.

### 2.5. Model Evaluation and Statistical Data Analysis

The approach to summing the number of chickens in the image was based on the “For loop and If” statement [33,34]. For loop structure allows code to be repeatedly executed to extract each center coordinates of bounding box. Several conditional statements were used to collect the population of chickens in the given area, normalize the frame of reference and add a bounding box to the object (chicken). When the input class (CLS) was 0, the code would generate an accumulator—a function that takes a number 1 and returns the total number incremented by 1.

In this study, precision, recall, F1 score and mean average precision (*mAP*) metrics were applied to assess the performance of the trained model in detecting chickens [32]. Detailed calculation processes are showed in equations below:

(1) $$Precision=\frac{TP}{\left(TP+FP\right)}$$

(2) $$Recall=\frac{TP}{\left(TP+FN\right)}$$

(3) $$F1\;score=\frac{\left(2*Precision*Recall\right)}{\left(Precision+Recall\right)}$$

(4) $$mAP=\frac{1}{n}\sum_{k=1}^{k=n}AP_{k}$$

where *AP_k_* is the average precision of the class *k* and *n* is the number of classes.

A true positive (*TP*) is a result where the model accurately concludes the positive class in chicken detection. Similarly, a false positive (*FP*) is an outcome where the model falsely predicts the positive class in chicken detection. A false negative (*FN*) indicates that the model incorrectly predicts the class. The mAP@0.5 indicates that it is the *mAP* calculated when at IOU (Intersection over Union) threshold 0.5.

A one-way ANOVA and Tukey HSD were conducted using JMP software (JMP Pro 16 for Mac, SAS Institute, Cary, NC, USA) to determine if there are significant differences between the predicted number of chickens and the actual number of chickens under different production and environmental conditions (i.e., light intensity, ages, stock density and observational angles) [35]. The difference was considered significant at *p* < 0.05.

## 3. Results and Discussion

### 3.1. Performance of the YOLOv5x-Hens

A confusion matrix of the best *YOLOv5x-hens* model was generated after training 300 epochs. The numbers of 970, 42, 46 and 0 were the true positive number, false positive number, false negative number, and true negative number, respectively. Table 2 sums up the results of performance metrics for *YOLOv5x-hens* and provides a comparison to the *YOLOv3,* a widely used CNN model. The target objects were chickens in both models, but the chickens’ feathers are presented differently. In the *YOLOv5x-hens*, the feathers are in white, but they are in yellow in *YOLOv3*. Additionally, the floor bedding/litter color in the two experiments was not the same. In our experiment, the color of litter was close to white, but it was brown in [29]. From an overview of these two models, they both performed well in target detection, but the *YOLOv5x-hens* has a higher recall by 8%, although the precision is 3% lower. For our newly developed model, our dataset is from white hens living on white bedding materials, which was more challenging in edge detection. In addition, our experiment was based on cage-free facilities, which contained more frequently moving birds that changed their positions so fast between adjacent frames and affected our training effectiveness [14]. Therefore, our model performed accurately in detecting the real-time number of birds from pullet (young hens) to layers (mature hens).

### 3.2. Convergence Results of Object Detector

Datasets were divided into training and verification sets, the loss curves consisted of loss of frame errors, and the loss of the hens on the floor (Figure 4). The frame loss is defined as the amount of service frames that are not delivered to their destination node. A high frame loss value indicates an unsatisfied prediction rate. The object loss is a compound loss based on the probability that object detection occurs in the region proposed. A high object loss means the accuracy of the model needed to be improved [36].

**Figure 5:**
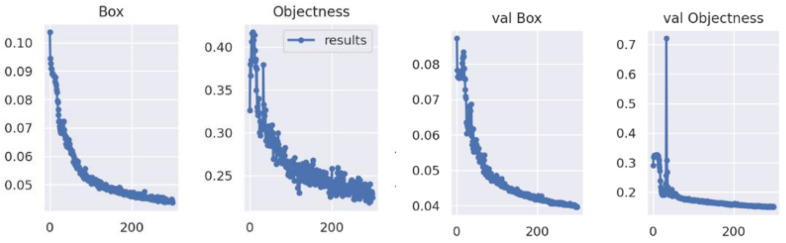
Convergence of the loss functions of training and validation sets (Box and Objectness are the frame loss and hens’ loss of training set respectively; val box and val objectness are the frame loss and hens’ loss of validation set, respectively).

From an overview in Figure 5, the loss function of the training and validation process showed a downward trend during the whole process, the Stochastic gradient descent (SGD) optimized the objective function with suitable paraments that correspond to the best fit between the predicted and actual outputs. Before the training batch reached 100, the loss values decreased rapidly, and the accuracy, precision and average accuracy upgraded rapidly. The SGD kept on iterating. When the training epochs arrived 200, the decreasing trend in the loss values slowed. Similarly, the improving parameters also slowed. When the training batch reached approximately 280, the loss function values of the training and validation sets showed a slight change, which indicates where the accuracy and precision of the model arrived at its peak. The best model weights were outputted after training finished.

### 3.3. Evaluation of Model Performance under Different Level of Light Intensity

**Figure 6:**
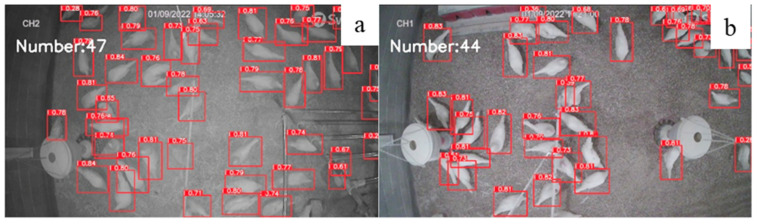
Number of chickens identified under different level of light intensity by our model: 10 lux (**a**) vs. 30 lux (**b**).

In the study, 200 photos were used to test the performance of *YOLOv5x-hens* under different levels of light intensity (10 lux and 30 lux; Figure 6). The accuracies at 10 lux and 30 lux are 95.15% and 95.02%, respectively. There is not a significant difference in accuracy between light intensity because deep learning, especially convolutional neural networks, display strong learning abilities due to inner detector algorithms, which apply a convolution layer, pooling layer and weight-based filter on each pixel of the image, enhancing the robust control [37].

### 3.4. Evaluation of Model Performance under Different Level of Flock Density

**Figure 7:**
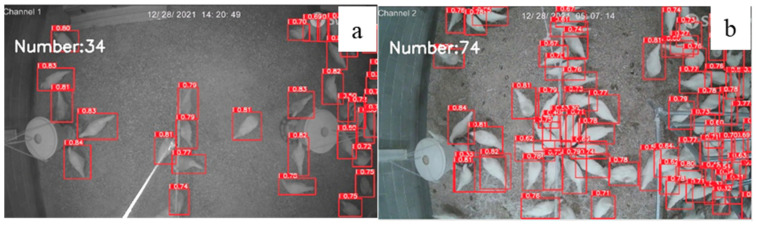
Number of chickens identified under low density and moderate density by our model: low density (**a**) vs. moderate density (**b**).

**Figure 8:**
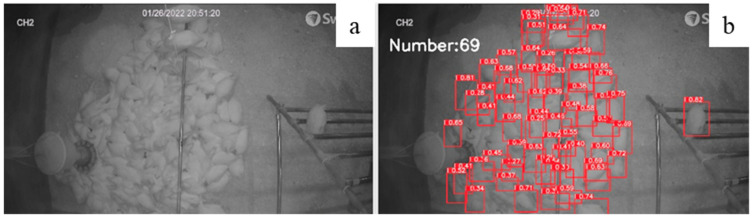
Number of chickens identified under high density by our model: original image of high density (**a**) vs. identified high density (**b**).

For model evaluation, 300 photos were used to test model accuracy under different levels of flock density (Figure 7 and Figure 8): low density (0–5 birds/m^2^), moderate density (5–9 birds/m^2^), and high density (9–18 bird/m^2^). For the three different densities, there was no difference in accuracies under low and middle densities (95.60% and 95.58%). Under the bird density of 9 birds/m^2^ or more, the accuracy of the model (60.74%) started to decrease due to extremely overlapped chickens and occlusion, which led to detector errors in classifying the hens’ boundaries [38]. Tracking individual hens from a pilling group is hard. In previous studies, the density map was used to estimate the chickens’ density, but the result tends to be unstable [39]. Therefore, further studies are needed to improve detector’s performance under high flock density.

### 3.5. Performance of YOLOv5x-Hens under Different Angles

**Figure 9:**
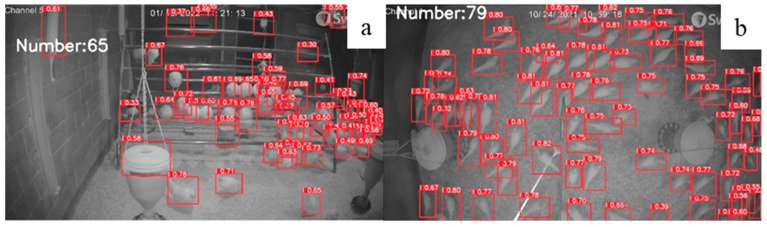
Number of chickens identified under horizontal angle and vertical angle by our model: horizontal angle (**a**) and vertical angle (**b**).

In the current study, cameras were installed on the celling (vertical angle) and sidewall (horizontal angle). A total of 200 images were used for evaluating the effect of angles on image quality. The model performance changed slightly under two different angels. It performed better in the vertical (96.33%) angle than the horizonal (82.24%) monitoring angle (Figure 9). Chickens could be occluded by feeders, drinking equipment and other facilities, which were previously noticed in broiler chicken houses [40,41]. Misidentifications were also observed from similar margins between chickens and other objects. In previous studies, the accuracy of vertical observation was also higher than that of horizonal observation. Researchers developed a region-based CNN model to detect chickens and reported that the model performed accurately under the vertical angle with 99.5% accuracy, while the accuracy of the horizontal angle was lover than 90% (i.e., 89.6%). For the horizontal angle, more objects tend to have similar margins to the target chickens (e.g., the shape of feeder is nearly same as the main body of a chicken) [40,41]. Therefore, collecting birds’ image data under a vertical angle is critical for developing a hens’ tracking model or system.

### 3.6. Performance of YOLOv5x-Hens under Different Ages of Birds

**Figure 10:**
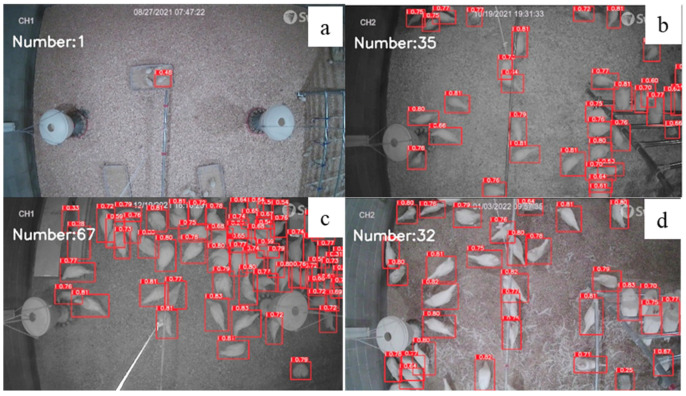
Number of chickens identified under different ages: (**a**) 1 week old, (**b**) 8 weeks old, (**c**) 16 weeks old, and (**d**) 18 weeks old with the newly developed model.

To test the accuracy of the *YOLOv5x-hens* under different stages of growth, video data collected at the birds’ age of week 1, week 8, week 16, and week 18, were used because these ages represent different key stages of laying hens (i.e., baby chick, teenage, first egg stage, and adult stage) (Figure 10). From teenage to first egg stage, the model performed accurately in detecting individual hens (around 96.0%). For baby chicks’ (<1 week) detection, however, the model achieved only about 25.6% accuracy due to the chicks’ small body size. Similar experiments have been conducted in broiler houses. A faster region-based CNN model was developed to detect broiler chickens continuously and showed stable performances for 4–5 weeks old broilers, which have reached market body weight (e.g., 2–2.5 kg) [14]. For cage-free houses, the monitoring accuracy of the *YOLOv5x-hens* was observed to be higher with the increase in birds’ age until week 16 or 17, when the pullets had a similar body size to matured hens.

## 4. Conclusions

In this study, a CNN-based deep learning model *YOLOv5x-hens* was built and evaluated to track hens (e.g., real-time number of hens in different locations) on the litter floor of cage–free facilities. The *YOLOv5x-hens* model performed efficiently in real-time detection under different lighting intensities, angles, bird densities, and ages over 8 weeks (i.e., birds were 8–16 weeks old). However, some misidentifications happened due to the hens’ pilling behaviors, uneven light intensities, and occluded images by equipment (i.e., drinking line and feeder). Future research will be guaranteed to address those issues (i.e., higher bird density with over 9 birds/m^2^) for improving model detection efficiency and applicability. The current study established the first real-time and accurate CNN model under cage-free facilities for the detection of pullets or hens. It provides a technical basis for developing a machine vision system for tracking individual pullets and hens for the evaluation of behavior and welfare status (i.e., sick birds or pododermatitis evaluation) in the future.

## Figures and Tables

**Table 1:** The difference between YOLOv5 models.

	*YOLOv5s*	*YOLOv5m*	*YOLOv5l*	*YOLOv5x*	Function
Depth_multiple	0.33	0.67	1.00	1.33	Model scaling [26]
Width_multiple	0.50	0.75	1.00	1.25	Model scaling [26]
ResNet in CSPNet	12	24	36	48	Computational loan [27]
Convolution kernel	512	768	1024	1280	Feature extraction [28]

**Table 2:** Performance metrics for *YOLOv5x-hens* and the comparison.

Items	Precision	Recall	F1 Score	mAP@0.5
*YOLOv3* [29]	0.988	0.875	0.926	/
*YOLOv5x-hens*	0.958	0.954	0.956	0.948

## Data Availability

Data available on request due to ethical restrictions.
